# Diffraction-limited imaging with monolayer 2D material-based ultrathin flat lenses

**DOI:** 10.1038/s41377-020-00374-9

**Published:** 2020-08-11

**Authors:** Han Lin, Zai-Quan Xu, Guiyuan Cao, Yupeng Zhang, Jiadong Zhou, Ziyu Wang, Zhichen Wan, Zheng Liu, Kian Ping Loh, Cheng-Wei Qiu, Qiaoliang Bao, Baohua Jia

**Affiliations:** 1 Centre for Translational Atomaterials, Faculty of Science, Engineering and Technology, Swinburne University of Technology, P. O. Box 218, Hawthorn, VIC 3122 Australia; 2 Department of Materials Science and Engineering, ARC Centre of Excellence in Future Low-Energy Electronics Technologies (FLEET), Monash University, Wellington Road, Clayton, VIC 3800 Australia; 3 School of Mathematical and Physical Sciences, Faculty of Science, University of Technology Sydney, 15 Broadway, Ultimo, NSW 2007 Australia; 4 Institute of Microscale Optoelectronics, Lab of Artificial Microstructure for Optoelectronics, Shenzhen University, 518000 Shenzhen, China; 5 School of Materials Science and Engineering, Nanyang Technological University, Singapore, 639798 Singapore; 6 Department of Chemistry, National University of Singapore, Singapore, 117543 Singapore; 7 Department of Electrical and Computer Engineering, National University of Singapore, Singapore, 117583 Singapore; 8 The Australian Research Council (ARC) Industrial Transformation Training Centre in Surface Engineering for Advanced Materials (SEAM), Swinburne University of Technology, P. O. Box 218, Hawthorn, VIC 3122 Australia

**Keywords:** Nanoparticles, Micro-optics

## Abstract

Ultrathin flat optics allow control of light at the subwavelength scale that is unmatched by traditional refractive optics. To approach the atomically thin limit, the use of 2D materials is an attractive possibility due to their high refractive indices. However, achievement of diffraction-limited focusing and imaging is challenged by their thickness-limited spatial resolution and focusing efficiency. Here we report a universal method to transform 2D monolayers into ultrathin flat lenses. Femtosecond laser direct writing was applied to generate local scattering media inside a monolayer, which overcomes the longstanding challenge of obtaining sufficient phase or amplitude modulation in atomically thin 2D materials. We achieved highly efficient 3D focusing with subwavelength resolution and diffraction-limited imaging. The high focusing performance even allows diffraction-limited imaging at different focal positions with varying magnifications. Our work paves the way for downscaling of optical devices using 2D materials and reports an unprecedented approach for fabricating ultrathin imaging devices.

## Optics: Lighting the way to atomically thin imaging devices

Ultrathin optical lenses made from monolayer two-dimensional transition metal dichalcogenides (TMDCs) could pave the way for next-generation imaging devices. The unique optical properties of monolayer TMDCs, including their large refractive indices in the visible range, make them ideal candidate for flat optical lenses. Although lenses made from multilayer TMDCs have been demonstrated, their insufficient phase or amplitude modulation from subnanometre thickness results in low focusing efficiencies of <1%. An international team, led by Professor Baohua Jia from Centre for Translational Atomaterials, Swinburne University of Technology, used femtosecond laser to generate nanoparticles for ultrathin lens on monolayered TMDC crystals. The lens has a subwavelength resolution and a high focusing efficiency of 31%, achieving diffraction limited imaging. Such an atomaterial lens lays the foundation for functional optical devices for applications in nano-optics and on-chip integration.

## Introduction

Ultrathin flat lenses with the ability to focus optical energy with minimal aberration have attracted great attention as essential optical components in nano-optics and on-chip photonic systems^1^. Recently, metasurfaces^2–9^, metamaterials^10^ and superoscillations^11^ have been developed to realize flat lenses with thicknesses of several tens to several hundreds of nanometres. According to the working principle of a dielectric metasurface lens, the axial and lateral dimensions (*d*) of the individual nanoscale elements should be around the effective wavelength (*λ*/*n*) of the incident light (*d* = *λ*/*n*, where *n* is the refractive index of the material) to introduce the desired phase and/or amplitude modulations. Thus it is challenging to further reduce the thickness of the lenses based on these principles. The ultimate thickness limit of a flat lens is a monolayer of atoms, which can be realized by using monolayer two-dimensional (2D) materials. However, when the material thickness is reduced to the subnanometre scale, the insufficient phase or amplitude modulation based on the intrinsic refractive index and absorption of the materials results in poor lens performance. Therefore, it is challenging to use ultrathin 2D materials to achieve sufficient phase or amplitude modulation in ultrathin flat lenses. The practical applications of such flat lenses for imaging require improving the efficiency and reducing the cost of production through (1) a new optical modulation strategy, (2) new growth methods to prepare materials with suitable dimensions and optical properties and (3) simple and scalable fabrication technologies.

2D layered materials, e.g. graphene and transition metal dichalcogenides^12^ (TMDCs) MX_2_, with M a transition metal atom (Mo, W, etc.) and X a chalcogen atom (S, Se or Te), have been intensively studied as candidates for next-generation nanometric optoelectronic devices due to their strong light–matter interactions resulting from 2D quantum confinement^13–18^. Moreover, the unique optical properties of monolayer TMDCs, namely, the extraordinarily large refractive indices in the visible range, can be leveraged for making flat lenses^19^. Flat lenses based on 200-nm-thick graphene oxide (GO) films can achieve highly efficient (~32%) three-dimensional (3D) focusing with a high resolution^20^ due to the significant refractive index and absorption modulation in laser-reduced GO. However, further reducing the thickness of GO will compromise the focusing efficiency and the resolution. Although optical lenses with multilayer graphene^21^ and TMDCs (MoS_2_)^22^ have been demonstrated, the focusing resolution is >10*λ* and the efficiency is <1% due to the limited phase and amplitude modulations. It remains a great challenge to further reduce the thickness of state-of-the-art flat lenses without sacrificing the focusing performance in terms of resolution and efficiency. Moreover, experimental demonstration of diffraction-limited optical imaging using ultrathin 2D material flat lenses, which is needed in the roadmap for miniaturized optical elements, has thus far been elusive due to the low resolution and efficiency.

Here we experimentally demonstrate a strategy to create a high-performance flat lens based on a monolayer TMDC single crystal with a thickness of approximately 7 Å, which corresponds to the physical thickness limit of the material. The key to achieving a milestone performance was to use direct femtosecond laser writing to pattern lens structures in the monolayer TMDC crystals through local generation of nanoparticles that strongly scatter the incident light to obtain desired amplitude and phase modulations. 3D focusing with a subwavelength lateral resolution (*λ*/2) and a high focusing efficiency of 31% was observed. More importantly, with such a high focusing performance, we were able to demonstrate the diffraction-limited imaging capability of a lens based on a monolayer van der Waals material for the first time.

## Results

**Fig. 1:**
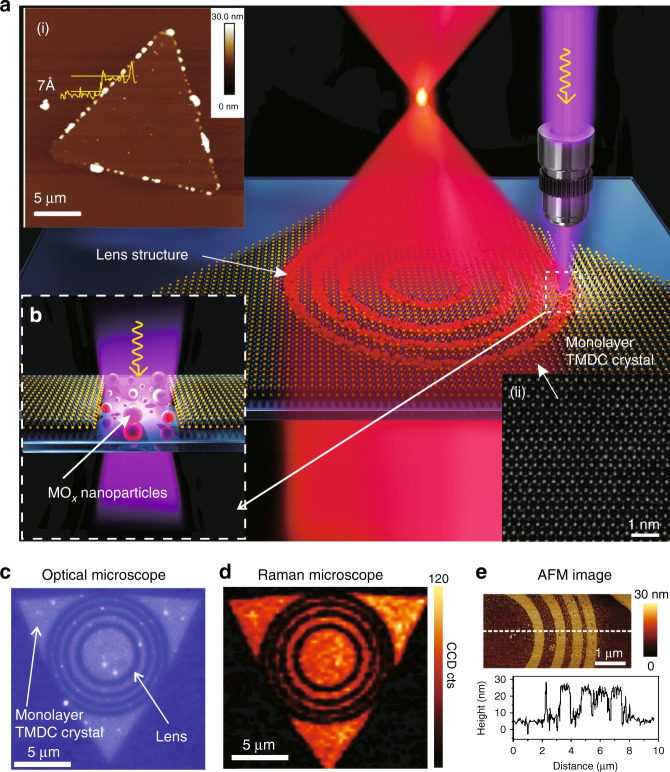
Laser fabrication of flat lenses in monolayer TMDC materials. **a** Schematic of femtosecond laser fabrication of a monolayer TMDC lens. Inset: (i) AFM image of a monolayer TMDC (WSe_2_ in this case) single crystal, and (ii) STEM image of the monolayer TMDC (WSe_2_ in this case) single crystal. **b** Schematic of femtosecond laser-induced generation of MO_*x*_ nanoparticles. **c**, **d** Optical microscopic image and Raman *E*^1^_2g_ band intensity image of a monolayer TMDC (WSe_2_ in this case) lens. **e** AFM image of the monolayer TMDC (WSe_2_ in this case) lens and the cross-sectional profile

The monolayer TMDC (i.e. WSe_2_) lens structure consisting of concentric rings was constructed by a femtosecond laser writing process^18^ (Fig. 1a), in which the femtosecond laser pulses were focused by a high numerical aperture (NA) objective lens (×100, 0.85 NA) onto the surface of an MX_2_ single crystal. A WSe_2_ single crystal was synthesized with an atmospheric pressure chemical vapour deposition (APCVD) system (Supplementary Section S1)^23^. The monolayer nature of the WSe_2_ crystal was verified by atomic force microscopy (AFM) (inset (i) in Fig. 1a) and scanning transmission electron microscopy (STEM) (inset (ii) in Fig. 1a; a high-resolution transmission electron microscopy (HRTEM) image is shown in Supplementary Section S1.2), and the thickness was confirmed to be ~7 Å. Here a femtosecond laser was applied for interaction with the TMDC material to create size-controlled nanoparticles. The dynamics of the interactions between the femtosecond pulses and WSe_2_ material are unique in two ways. First, the extremely short pulse duration in time translates into very high peak intensities (on the order of GW m^−2^) that can drive nonlinear and multiphoton absorption processes^24^. Second, the pulse delivers energy to the material on a timescale shorter than the electron–phonon coupling relaxation time, where the latter is estimated to be in the range of nanoseconds^25^. Thus the incident pulse only delivers energy to the electrons, leaving the ions completely “cold”^25^. Under such circumstances, the W-Se bonds are broken due to the photochemical effect^26^, leaving W^4+^ ions with free bonds, which react with oxygen in air, forming WO_*x*_ nanoparticles on the substrate (Fig. 1b), as described by the following equation:

1 $${\rm{WSe}}_2 + {\rm{O}}_2 \to {\rm{WO}}_x + {\rm{SeO}}_x$$

The formation of WO_*x*_ was verified by scanning X-ray photoelectron spectroscopy (XPS) measurements (Fig. S4). The process is significantly different from continuous wave laser ablation, in which ablation is enabled by heating the entire lattice and no nanoparticles are generated^27^.

The height and lateral size of the particles are approximately 20 and 50–150 nm, respectively, as measured by AFM (Fig. 1e and Fig. S2a, b), which are in the range of Rayleigh scattering^28^. This scattering decreases the direct transmission and reflection from the material, which can be clearly seen from the optical microscopic image (Fig. 1c) and was also observed during the laser fabrication process (Supplementary Movie S1). By using confocal Raman mapping (see Fig. 1d), the intensity of the characteristic band (*E*^1^_2g_, also see the Raman spectra in Supplementary Fig. S5) in the laser-treated areas is found to be extremely low, suggesting the removal of WSe_2_ material.

**Fig. 2:**
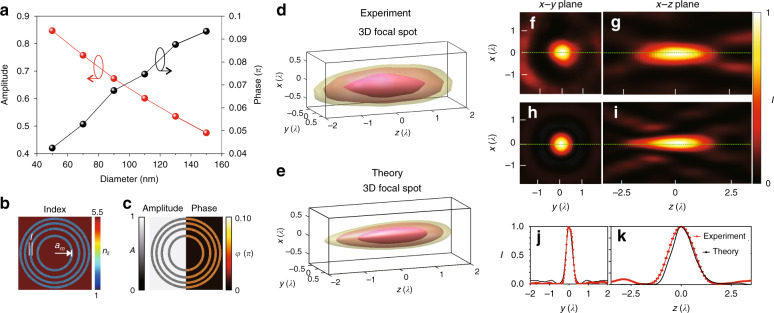
Focusing of optical energy using a monolayer TMDC material lens. **a** Phase and amplitude modulations arising from WO_2_ nanoparticles with different diameters. **b** Simulated index profile of the WSe_2_ lens. **c** Simulated amplitude and phase distributions of the WSe_2_ lens. **d** 3D focal spot from experimental measurement. **e** 3D focal spot from theoretical simulation. Cross-sectional images of the focal spot from the experiment (**f**, **g**) and simulation (**h**, **i**). Cross-sectional intensity plots along the *y*-direction (**j**) and *z*-direction (**k**)

The finite-difference time-domain (FDTD) method was used to calculate the phase and amplitude modulations provided by nanoparticles with a range of sizes. By knowing the phase and amplitude modulations, a theoretical model based on Rayleigh–Sommerfeld diffraction theory was developed to design and simulate the intensity distributions in the focal region of the lens, which is a much faster approach than FDTD simulations. In this way, we can theoretically simulate the intensity distribution of the designed lenses in the focal region with high accuracy. The ranges of the amplitude and phase modulations arising from the scattering were calculated by using the FDTD method based on the assumption that the nanoparticles are ellipsoid in shape and have random lateral sizes ranging from 50 to 150 nm (Fig. 2a). The complex permittivity (including the real and imaginary parts) of the material was obtained by measuring a commercial WO_2_ sample using spectral ellipsometry (Supplementary Fig. S6), which shows platinum-like metallic properties as reported^29^. It is noteworthy that a 0.85 amplitude (85% of the light incident on the nanoparticles is scattered) and a 0.1 *π*-phase modulation can be achieved due to the scattering (Fig. 2a). Then an analytical model based on Rayleigh–Sommerfeld diffraction theory^28^ was developed to evaluate the focusing capability of the WSe_2_ lens, in which the spatial distribution of the refractive index is shown in Fig. 2b (the refractive index of the monolayer WSe_2_ material is 5.5 at the wavelength of 633 nm, Supplementary Fig. S7) considering that particles with random sizes are randomly positioned along the ring and the coverage of the particles in the area is approximately 80%. The corresponding phase and amplitude modulations are shown in Fig. 2c. There are two key parameters for achieving high-performance lens design, namely, the position (*a*_*m*_) and the width (*l*) of each ring (Fig. 2b). Here the width is determined by the laser fabrication linewidth, which is approximately 400 nm (see Supplementary Section S2 and Fig. S3). The position of each ring is determined by using the Rayleigh–Sommerfeld method described in our previous study^30^ considering the incident wavelength (*λ*) and the desired focal length (*f*). In general, the method can be used to design flat lenses with arbitrarily focal lengths and diameters. This method overcomes the limitation of the Fresnel lens design method, in which the spacing between neighbouring rings decreases with increasing number of rings. Therefore, when the spacing becomes smaller than the fabrication resolution limit, the rings cannot be separated and contribute to the focusing. Thus the size of the lenses from Fresnel design is limited. In contrast, the Rayleigh–Sommerfeld method can be used to optimize the position of each ring by calculating the focusing performance, in which the required spacing between each ring can be set as a criterion. In this way, it is possible to design large flat lenses with high NA. The effective NA of the lens depends on the number of rings (*N*). The overall radius of the lens is defined by the radius of the outermost ring (*a*_*N*_). Therefore, the effective NA can be calculated as NA = *a*_*N*_/*f*.

One example of the calculated 3D focal intensity distributions of a lens (*N* = 5, *f* = 14 μm) is shown in Fig. 2e, and the cross-sectional plots in the lateral and axial directions are shown in Fig. 2h, i, respectively. A well-defined strong focus with ignorable sidelobes (<10% of the intensity of the central lobe) is observed. The full width at half maximum (FWHM) of the focal spot in the lateral direction (*W*_*x*_) is 0.51*λ* and that in the axial direction (*W*_*z*_) is 1.54*λ*, leading to a high-quality 3D focal spot with wavelength-scale resolution. This *W*_*z*_ is slightly longer than that of the normal refractive objective lens (~1.25*λ*) with the same lateral resolution. This might be induced by the amplitude modulation mechanism provided by the nanoparticles, which minimizes the light from the zone contributing to destructive interference. Compared to the continuous phase modulation provided by refractive objective lenses with a curved surface, amplitude modulation is less effective in achieving the optimal interference condition to minimize the FWHM along the *z*-direction. This FWHM can be further improved in the future by maximizing the phase modulation arising from the nanoparticles and arranging the phase modulation according to the optimal interference condition. The corresponding experimental demonstration was performed using a confocal scanning optical microscope (Supplementary Fig. S8), in which the 2D cross-sectional focal spot distribution was imaged in series with a step of 10 nm in the axial direction (Supplementary Movie S2). A strong 3D focus can be clearly identified (Fig. 2d), which reveals a subwavelength focusing performance in the lateral dimensions (Fig. 2f) and a high axial resolution (Fig. 2g). A comparison between the experimental and theoretical results is shown in Fig. 2j, k, and good agreement can be observed. The focusing efficiency is defined as the ratio of the total power in a circular aperture with a radius three times the FWHM of the focal spot to the total power of light transmitted through the lens^9^, which is approximately 31%. To further confirm the role of nanoparticles, a similar lens structure was fabricated from the same material by a focused ion beam (FIB) process as a comparison. Very few nanoparticles were observed in the FIB-milled area after the optimized FIB fabrication process (Supplementary Fig. S9a), which was also confirmed using AFM (Supplementary Fig. S9b, c). It is found that such a lens cannot provide sufficient optical modulation of incident light; thus no focusing can be achieved. Here the lens can work for a single wavelength (with a small wavelength range of 50 nm) to achieve the optimal focusing condition through constructive interference of the light at the focal position, although the nanoparticles can scatter light of different wavelengths with different modulation strengths. Therefore, different lens designs must be developed to target light focusing at different wavelengths. With the current design, it is expected that the focusing efficiency at different wavelengths will drop significantly to only a few percent due to the less optimal interference condition.

**Fig. 3:**
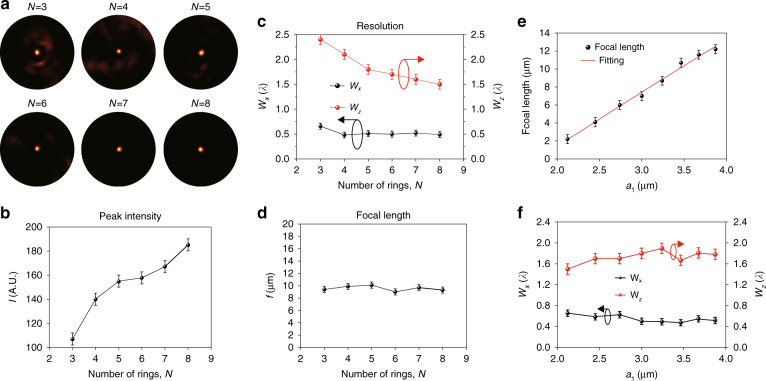
Focusing characterization of different lens designs. **a** Images of foci of lenses with different numbers of rings (*N* = 3–8). **b** Plot of the peak intensity of the focus versus *N*. **c** Resolution of the focus versus *N*. **d** Focal length versus *N*. **e** Focal length versus *a*_1_. **f** Resolution versus first ring radius *a*_1_

To further evaluate the focusing capability of the WSe_2_ lens, the dependence on the number of rings (*N*) was investigated (*N* was varied from 3 to 8). It is found that the contrast of the focus to the background is improved by increasing *N* (Fig. 3a) since more light constructively interferes at the focus with more rings. Controlled experiments suggest that the lens requires at least three rings to achieve decent focusing. The peak intensity of the focal spot increases as *N* increases, as depicted in Fig. 3b. The FWHMs along the lateral (*W*_*x*_) and axial (*W*_*z*_) directions are shown in Fig. 3c. The detailed information can be found in Supplementary Table S1. A subwavelength lateral resolution (approximately 0.5*λ*) can be achieved when *N* is >5. In addition, the axial resolution can be continuously improved by increasing the number of rings, as the light is strongly diffracted by the outer rings, which results in a large convergence angle *β*. The wave vector along the axial direction is *k*_*z*_ = *k*cos*(β)*, where *k* = *2π*/*λ* is the wave vector of light in free space. Therefore, a large convergence will result in a smaller *k*_*z*_, which further contributes to a higher focusing resolution.

We observed that the focal length *f*, which is determined by *a*_*m*_ (*m* = *1*,*2*,*…*,*N*), remains almost unchanged while varying *N*, as shown in Fig. 3d. According to our theoretical model^30^, the radius of the innermost ring can be expressed as $a_1 = \sqrt {\lambda f}$ (*λ* is the incident wavelength, which is 633 nm here), and we fabricated lenses consisting of five rings (*N* = 5) with *a*_1_ ranging from 1.37 to 3.89 µm. When *a*_1_ is <2 µm, the peak intensity of the focal spot is too low to be distinguished from the background due to the small light collection area of the lens. The resulting dependence of *f* on *a*_1_ is shown in Fig. 3e, which can be fitted with a parabolic function, confirming the accuracy of our theoretical model. The FWHMs along the lateral (*W*_*x*_) and axial (*W*_*z*_) directions are plotted in Fig. 3f. It is found that the FWHMs are almost unchanged against *a*_1_ since the effective NA of the lenses depends only on the number of rings (*N*).

**Fig. 4:**
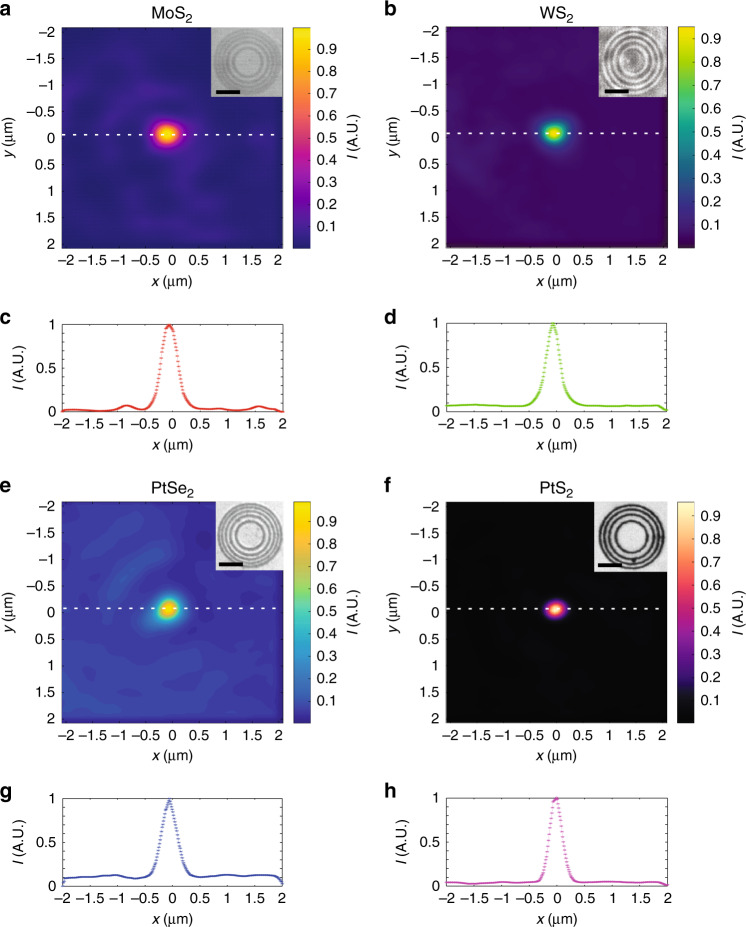
Ultrathin flat lenses of different monolayer TMDC materials. Experimentally measured focal intensity distribution of lenses of different monolayer TMDC materials: **a**, **c** MoS_2_, **b**, **d** WS_2_, **e**, **g** PtSe_2_, and **f**, **h** PtS_2_. The white dashed lines mark the corresponding locations of the intensity cross-sectional plots along the *x*-direction shown in **c**, **d**, **g**, **h**. Inset: reflective optical microscopic images. Scale bar: 5 μm

For unambiguous verification of the working principle of the lens based on a monolayer TMDC material, we fabricated lens structures using other TMDC materials, including MoS_2_, WS_2_, PtSe_2_ and PtS_2_, as shown in Fig. 4. The concentric ring structure can be clearly identified in the optical microscopic images, as depicted in the insets of Fig. 4a, b, e, f. All the lenses are capable of focusing light with high resolution and high efficiency, as shown in the intensity contour plots (Fig. 4a, b, e, f). The cross-sectional intensity plots (Fig. 4c, d, g, h) show that the FWHMs of all the focal spots of the different lenses are in the subwavelength region, ranging from 0.43*λ* to 0.55*λ* (Supplementary Fig. S10). In addition, the sidelobes can be controlled to a low level (<0.2 of the central peak intensity), confirming that most of the optical energy is efficiently concentrated at the focal spots. These results not only corroborate the high accuracy of the theoretical model but also imply that the concept of a lens fabricated in a monolayer TMDC material by femtosecond laser writing can be generally applied to other TMDC materials. Although the same approach can be generally applied to different types of TMDC materials, the properties of the generated nanoparticles are different depending on the original TMDC materials. The amount of amplitude modulation provided by the nanoparticles is proportional to the contrast of the rings in the optical microscopic images in the inset of Fig. 4. The higher the optical contrast is, the larger the amplitude modulation and thus the better the focusing performance that can be achieved. The PtS_2_ material presents the highest contrast; thus the PtS_2_ lens achieves the highest focusing performance, as shown in Fig. 4 and Supplementary Fig. S10. Enabled by the high flexibility and reproducibility of the femtosecond laser writing method, one can design and fabricate lenses with desired focal length *f*, number of rings *N* and NA based on different TMDC materials to meet the requirements of different applications using the theoretical model and femtosecond laser writing method. The NAs of the TMDC lenses versus different parameters, including the number of rings *N* and the radius of the innermost ring *a*_1_, are shown in Fig. S8. As shown in the figure, for a given focal length (*f* = 9 μm in the plot), the NA can be effectively increased from 0.46 to 0.99 by increasing *N* from 3 to 14 and can be further increased by simply increasing *N*. This shows the flexibility of the design method, and *N* can be optimized according to the required NA. On the other hand, the NA decreases with increasing *a*_1_ for a given *N* because *f* increases faster than the overall size of the lenses (*a*_*N*_). Therefore, it is necessary to increase *N* to maintain the NA of the lens when *f* is increased.

**Fig. 5:**
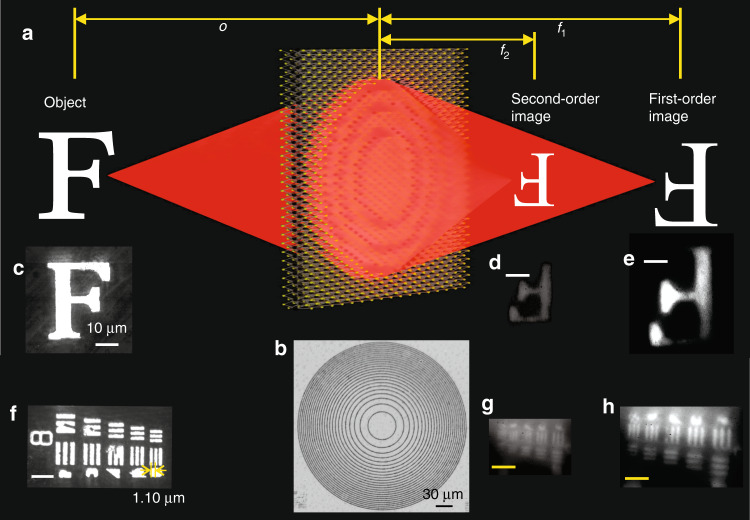
Diffraction-limited imaging using a monolayer TMDC lens. **a** Schematic illustrating diffraction-limited imaging using a monolayer TMDC lens. **b** Optical microscopic image of a large-scale monolayer TMDC lens (300 μm in diameter, *f*_1_ = 300 μm). **c** Optical microscopic image of the object letter “F”. **d** Second-order image of the object “F”. **e** First-order image of “F”. **f** Optical microscopic image of the USAF standard board. **g**, **h** Second- and first-order images of the USAF standard board. The scale bar in **d**, **e**, **g**, **h** is 10 μm

As imaging is one of the essential functions of a lens, we further validated the imaging capability of a lens based on a TMDC thin film (see Fig. 5). A schematic of the imaging process is shown in Fig. 5a. A lens with a diameter of 300 μm was fabricated in a large PtS_2_ film, as shown in Fig. 5b. Practically, the images from the lens were further magnified by a 4*f* microscopic imaging system and collected using a charge-coupled device (CCD) camera (Supplementary Section S9 and Figs. S11 and S12). As a diffractive lens with a focal length (300 μm) much larger than the incident light wavelength (white light illumination in the visible region centred at ~550 nm) was used, it is possible to have multiple focal lengths corresponding to the diffraction orders, namely, *f*_1_ and *f*_2_ in this study for the first and second diffraction orders (Fig. 5a). This leads to multiple images at different locations without crosstalk, which effectively gives the lens an optical zoom capability based on the lens law of a diffractive lens (Supplementary Section S10):

2 $$\frac{1}{o} + \frac{1}{i} = \frac{2}{f}$$

where *o* is the distance between the object and the lens, while *i* is the distance from the image to the lens. The magnification rate of the lens is *M* = *i*/*o*. By tuning the focal length, the distance of the image and the magnification rate can be tuned accordingly. A higher diffraction order results in shorter focal length and image distance, thus giving a smaller magnification rate.

Images with high contrast and spatial resolutions of two objects (the letter “F” in Fig. 5c (Supplementary Movie S3) and the USAF (U.S. Air Force) standard target in Fig. 5f (Supplementary Movie S4)) can clearly be identified in Fig. 5d, e, g, h (a detailed explanation and the image properties can be found in Supplementary Materials). The minimal distance between bars in the USAF standard target board in this experiment is 1.1 μm (Fig. 5f), which can be clearly identified in the first-order image (Fig. 5h), confirming the achievement of diffraction-limited in-plane imaging. Although the first-order diffraction is currently stronger than the second-order diffraction based on our design, it is possible to design a lens with arbitrary focusing strengths for different orders based on the optimization method^31^ to meet the specific requirements. In addition, the strategy is easily accessible, as monolayer TMDC crystals can be transferred onto arbitrary substrates with a nondestructive polystyrene-mediated transfer technique^23^, making our lens readily integrable with diverse electronic or photonic devices to realize imaging, sensing, energy harvesting and communication functions.

## Discussion

In conclusion, we fabricated an ultrathin flat lens using a monolayer TMDC crystal that is capable of 3D subwavelength focusing and diffraction-limited imaging in the far field. A key aspect of our work is the use of femtosecond laser patterning to create strong scattering centres in the monolayer TMDC; these centres further provide amplitude and phase modulations that overcome the inherent thickness limit of the monolayer and lead to a high focusing efficiency and 3D diffraction-limited focusing. The focusing properties of the lenses can be tuned by controlling the radius and number of rings. Furthermore, the diffraction-limited imaging capability was demonstrated by imaging a USAF standard target using a large-scale lens. Although other ultrathin flat lenses^32,33^ with similar deep-subwavelength overall thicknesses (tens of nanometres) based on plasmonic or Fresnel zone plate principles have been previously demonstrated using a multiple-step nanofabrication process involving electron beam lithography and ion etching, imaging functionality has not been achieved. These complex and high-cost processes have proven unsuitable for lens fabrication in monolayer materials. The demonstrated ultralightweight, highly efficient and integration-ready flat lenses based on monolayer 2D materials open up new avenues for wide-ranging photonic applications and miniaturized lab-on-chip devices. In addition, the demonstration of multiorder images suggests that it is possible to create a flat zoom lens without any physical moving components by simply using images from different diffraction orders with different focal lengths.

As the nanoparticles can scatter light of different wavelengths, it is possible to design an achromatic metalens by further developing the design method. One of the potential solutions is to achieve similar phase modulations at different wavelengths^34^. In this way, light of different wavelengths can be efficiently focused at the same position to create an achromatic lens for broadband optical imaging. In this study, nanoparticles are used to provide amplitude modulation, which decreases the overall transmission of light of the lens. Thus the efficiency can be further improved by exploring the potential to achieve phase modulation by using nanoparticles.

## Materials and methods

### Synthesis and characterization of van der Waals materials

#### Synthesis of WSe_2_

Monolayer WSe_2_ crystals were grown on SiO_2_ (quartz) and SiO_2_/Si substrates via APCVD^23^. The uniform contrast in individual crystals suggests an optically flat surface, and the monolayer crystals were nearly equilateral triangles with a side length of ~19 µm on quartz and even ~69 µm on SiO_2_/Si substrates^35^. The topography of the WSe_2_ flakes grown on quartz substrates was investigated with AFM. The whole crystal was nearly atomically flat with a SiO_2_/WSe_2_ step of 7 Å, confirming the thickness to be monolayer.

#### Synthesis of MoS_2_ and WS_2_

Mixed powder of NaCl (0.5 mg) and MoO_3_ (or WO_3_) (3 mg) in an alumina boat was placed in the centre of a quartz tube. The furnace was heated to the growth temperature (600–800 °C) with a ramp rate of 50 °C min^−1^. The growth time was 3–5 min. Ar (or Ar/H_2_) with a flow rate of 80 (80/5) sccm was used as the carrier gas^12^.

#### Synthesis of PtS_2_ and PtSe_2_

Pt layers of different thicknesses were sputter-coated onto SiO_2_/Si substrates. The Pt samples were sulfurized (selenized) in a quartz tube with a 1-inch diameter. Pt samples were loaded in the primary heating zone and heated to 400 °C (or 550 °C) for the growth of PtS_2_ (or PtSe_2_). The S (Se) source was loaded in the upstream zone, which was heated to the melting point of S (or Se). Ar/H_2_ (9:1), with a flow rate of 150 sccm, was used to transport the vapourized S (or Se) to the Pt samples. A dwell time of 0.2 h was used to ensure complete reaction^36^.

#### XPS characterization of the WSe_2_ material

XPS was used to measure the binding energy of W and Se. Supplementary Fig. S1a, b show the binding energy profiles for W 4f and Se 3d, respectively. The two peaks at 33.19 and 35.35 eV are attributed to W 4f_7/2_ and W 4f_5/2_ for WSe_2_, respectively. The other two peaks located at 36.12 and 38.99 eV are attributed to W 4f_7/2_ and W 4f_5/2_ for WO_*x*_, respectively. The peaks with binding energies of 55.40 and 56.25 eV can be assigned to Se 2d_5/2_ and Se 2d_3/2_, respectively. All these results are consistent with the reported values for the WSe_2_ crystal. The positions of these XPS peaks suggested that the valence of W is +4, which is evidence of the formation of the WSe_2_ phase^37–39^.

#### TEM characterization of the WSe_2_ material

HRTEM was also employed to investigate the microstructure and crystallinity of the monolayer WSe_2_ crystals. Low-magnification and HRTEM images of monolayer WSe_2_ are depicted in Supplementary Fig. S2a, b, respectively. The low-magnification TEM image in Fig. S2a shows a corner of a monolayer WSe_2_ crystal, where the contrast is relatively uniform. Fig. S2b shows the HRTEM image, where the atomic lattice of monolayer WSe_2_ is clearly resolved.

### Laser fabrication of flat lenses

A femtosecond laser beam (Libra, 100 fs pulse, 10 kHz repetition rate, 800 nm wavelength) that passed through a dichroic mirror was focused by a high NA objective lens (×100, NA = 0.85) onto a WSe_2_ sample, which was mounted on a 3D nanometric piezoelectric stage (PhysikInstrumente^®^). A computer-controlled system was used to control the parameters of the laser fabrication process, including the laser power, scanning speed and patterns. The threshold power was found to be 5 µW, corresponding to a pulse energy of 0.7 nJ. The circles started to overlap when the power was >14 µW (1.4 nJ pulse energy). These two powers defined the lower and upper limits of the laser power range, which was explored in 1 µW steps. Since the laser focal spot has a FWHM of 600 nm, the laser energy density in the centre of the focal spot varies between 0.2 and 0.56 J cm^−2^. The scanning speed was 10 µm s^−1^ to ensure smooth line fabrication.

### Characterization of femtosecond laser-written material

#### Scanning micro-XPS characterization of the femtosecond laser-written area

Scanning XPS measurements were conducted at the Synchrotron Radiation Research Center, Taiwan, China (SPEM end station of beamline 09A1). The soft X-ray beam (photon energy = 400 eV) was focused with Fresnel zone-plate optics to achieve a spatial resolution of 100 nm. Scanning photoelectron microscopic images were taken by scanning the sample holder on a piezo stage. The photon energy was routinely calibrated with the core-level line of Au at a binding energy of 84 eV. The overall energy resolution was better than 100 meV, and the experiments were conducted at room temperature.

#### Raman spectra of monolayer WSe_2_ material after milling

The complete removal of the WSe_2_ material in the patterned area was confirmed by Raman spectroscopy (Supplementary Fig. S5) and *E*^1^_2g_ band intensity imaging (inset Supplementary Fig. S5). The shrinkage of the *E*^1^_2g_ band at approximately 250 cm^−1^ and the rising peak at 308 cm^−1^ suggest that WSe_2_ is converted to WO_*x*_ after laser milling.

### Imaging system and focal spot measurement

A homemade imaging characterization system was built to study the performance of lenses, as schematically illustrated in Supplementary Fig. S8. The cross-sectional distributions of the generated focal spots of the WSe_2_ lenses (which were attached to SiO_2_ substrates) were captured using a CCD camera (Watec 902H3 SUPREME) equipped with a ×100 objective (NA = 0.85) and a tube lens (*f* = 200 mm). The lenses were illuminated by a collimated He-Ne laser at a wavelength of 633 nm. The magnification rate of the 4*f* image system was 110. A schematic of the imaging process is shown in Supplementary Fig. S12, in which the images from the TMDC lens were further magnified by a 4*f* microscopic imaging system composed of an objective (NA = 0.8, ×100) and a tube lens (*f* = 200 mm) and collected using a CCD camera (Watec 902H3 SUPREME). The objective lens was scanned along the axial direction to obtain images at different positions. The object was illuminated by a white light source (Philips Essential 35 W GU10 Dichroic Halogen Globe).

### Theoretical model and numerical simulation

The complex refractive indices (complex permittivities) of WSe_2_ and WO_2_ materials (Figs. S6 and S7) were measured by using a spectral ellipsometer (M-2000 J.A. Woollam Co) and fitted by using the built-in software (Complete Ease) based on Kramers–Kronig analysis^40^. The ranges of the amplitude and phase modulations due to the scattering were calculated by using the commercial FDTD software (Lumerical FDTD) based on the assumption that the nanoparticles were ellipsoid in shape and had random lateral sizes ranging from 50 to 150 nm. The lenses were theoretically designed using an analytical model based on Rayleigh–Sommerfeld diffraction theory^28^ and programmed using MATLAB. In our simulation, we consider a collimated plane wave incident on the lens with uniform phase and amplitude before being modulated by the lens.

## Supplementary information

Supplementary Information

Supplementary Movie S1

Supplementary Movie S2

Supplementary Movie S3

Supplementary Movie S4

## Data Availability

The data that support the plots within this paper and other findings of this study are available from the corresponding authors upon reasonable request.

## Code availability

The code used to calculate the results for this work is available from the corresponding author upon reasonable request.
