# Supplementary material for: Diffraction-limited imaging with monolayer 2D material-based ultrathin flat lenses
Source: Light Sci Appl. 2020 Aug 11;9:137. doi: 10.1038/s41377-020-00374-9 (PMC7421448; doi:10.1038/s41377-020-00374-9)
Supplement: Supplementary file 1 — Supplementary Information [file 41377_2020_374_MOESM1_ESM.docx]

Supplementary information for

Diffraction-limited imaging with monolayer 2D material-based ultrathin flat lenses

Han Lin^1^, Zai-Quan Xu^2,3^, Guiyuan Cao^1^, Yupeng Zhang^4^, Jiadong Zhou^5^, Ziyu Wang^2^, Zhichen Wan^2^, Zheng Liu^5^, Kian Ping Loh^6^, Cheng-Wei Qiu^7*^, Qiaoliang Bao^2*^, Baohua Jia^1,8*^

^1^Centre for Translational Atomaterials, Faculty of Science, Engineering and Technology, Swinburne University of Technology, P. O. Box 218, Hawthorn VIC 3122, Australia

^2^ Department of Materials Science and Engineering, ARC Centre of Excellence in Future Low-Energy Electronics Technologies (FLEET), Monash University, Wellington Road, Clayton, Victoria 3800, Australia

^3^School of Mathematical and Physical Sciences, Faculty of Science, University of Technology Sydney, 15 Broadway, Ultimo, 2007, NSW, Australia

^4^Institute of Microscale Optoelectronics, Lab of Artificial Microstructure for Optoelectronics Shenzhen University, Shenzhen 518000, China

^5^School of Materials Science and Engineering, Nanyang Technological University, Singapore 639798, Singapore

^6^Department of Chemistry, National University of Singapore, Singapore 117543, Singapore

^7^Department of Electrical and Computer Engineering, National University of Singapore, Singapore 117583, Singapore

^8^The Australian Research Council (ARC) Industrial Transformation Training Centre in Surface Engineering for Advanced Materials (SEAM), Swinburne University of Technology. PO Box 218, Hawthorn, VIC 3122, Australia

E-mail: [chengwei.qiu@nus.edu.sg](mailto:chengwei.qiu@nus.edu.sg); [qiaoliang.bao@gmail.com](mailto:qiaoliang.bao@gmail.com); [bjia@swin.edu.au](mailto:bjia@swin.edu.au)

# S1 Synthesis and characterization of van der Waals materials

## S1.1 Synthesis of van der Waals materials

**Synthesis of WSe_2_**: the monolayer WSe_2_ crystals are grown on a SiO_2_ (quartz) and SiO_2_/Si substrate *via* atmospheric pressure chemical vapour deposition (APCVD)^1^. The uniform contrast from individual crystal suggests an optically flat surface and the monolayer crystals are nearly equilateral triangular with a side length of ~19 µm on quartz and even ~69 µm on SiO_2_/Si substrate.^2^ The topography of the WSe_2_ flakes grown on quartz substrates is investigated with the atomic force microscopy (AFM). The whole crystal is nearly atomically flat with a SiO_2_/WSe_2_ step of 7Å, confirming the thickness to be monolayer.

**Synthesis of MoS_2_ and WS_2_**: Mixed powder of NaCl (0.5 mg) and MoO_3_ (or WO_3_) (3 mg) in alumina boat was placed in the center of the quartz tube. The furnace was heated to the growth temperature (600 to 800 ^o^C) with a ramp rate of 50 ^o^C/min. The growth time is 3 to 5 min. The Ar (or Ar/H_2_) with a flow rate of 80 (80/5) sccm was used as the carrier gas^3^.

**Synthesis of PtS_2_ and PtSe_2_**: Pt layers of diﬀerent thicknesses were sputter-coated onto SiO_2_/Si substrates. The Pt samples were sulfurized (selenized) in a quartz tube with 1-inch diameter. Pt samples were loaded in the primary heating zone and heated to 400 °C (or 550 °C) for the growth of PtS_2_ (or PtSe_2_). The S (Se) source was loaded in the upstream zone, which was heated to the melting point of S (or Se). Ar/H_2_ (9:1), with a ﬂow rate of 150 sccm, was used to transport the vaporized S(or Se) to the Pt samples. A dwell time of 0.2 h was used to ensure complete reaction^4^.

## S1.2 XPS characterization of the WSe_2_ material


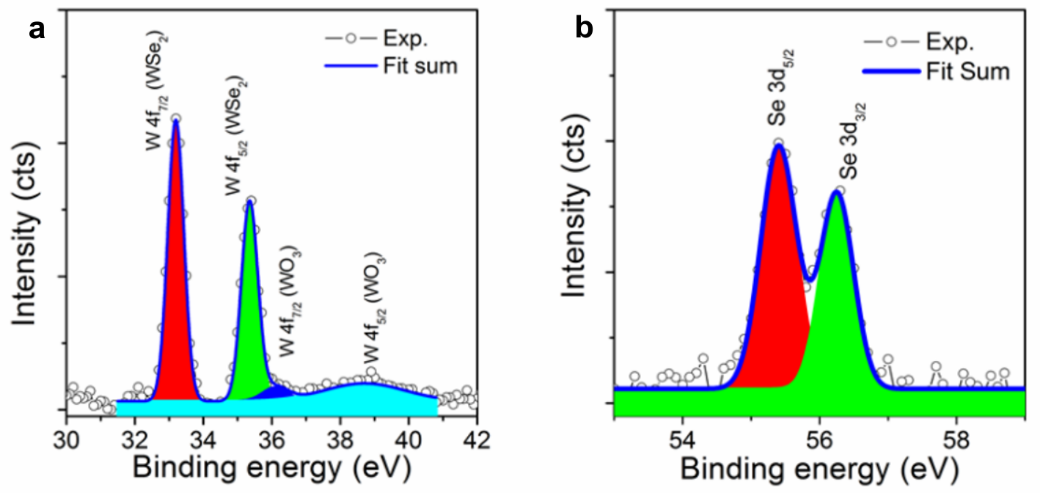


**Fig. S1** The binding energy profiles for W 4f and Se 3d, respectively. Two peaks at 33.19 eV and 35.35 eV are attributed to the W 4f_7/2_ and W 4f_5/2_ for WSe_2_, respectively. And the other two peaks located at 36.12 eV and 38.99 eV are attributed to the W 4f_7/2_ and W 4f_5/2_ for WO_x_, respectively. The peaks with binding energy of 55.40 eV and 56.25 eV can be assigned to the Se 2d_5/2_ and Se 2d_3/2_, respectively.

In order to study the chemical composition of as-prepared WSe_2_, X-ray photoemission spectroscopy (XPS) was used to measure the binding energy of W and Se. Fig. S1a and b show the binding energy profile for W 4f and Se 3d, respectively. Two peaks at 33.19 eV and 35.35 eV are attributed to the W 4f_7/2_ and W 4f_5/2_ for WSe_2,_ respectively. And the other two peaks located at 36.12 eV and 38.99 eV are attributed to the W 4f_7/2_ and W 4f_5/2_ for WO_x_, respectively. The peaks with binding energy of 55.40 eV and 56.25 eV can be assigned to the Se 2d_5/2_ and Se 2d_3/2_, respectively. All these results are consistent with the reported values for WSe_2_ crystal. The positions of these XPS peaks suggested that the valence of W is +4, an evidence for the formation of WSe_2_ phase.^5-7^

## S1.3 TEM characterization of the WSe_2_ material


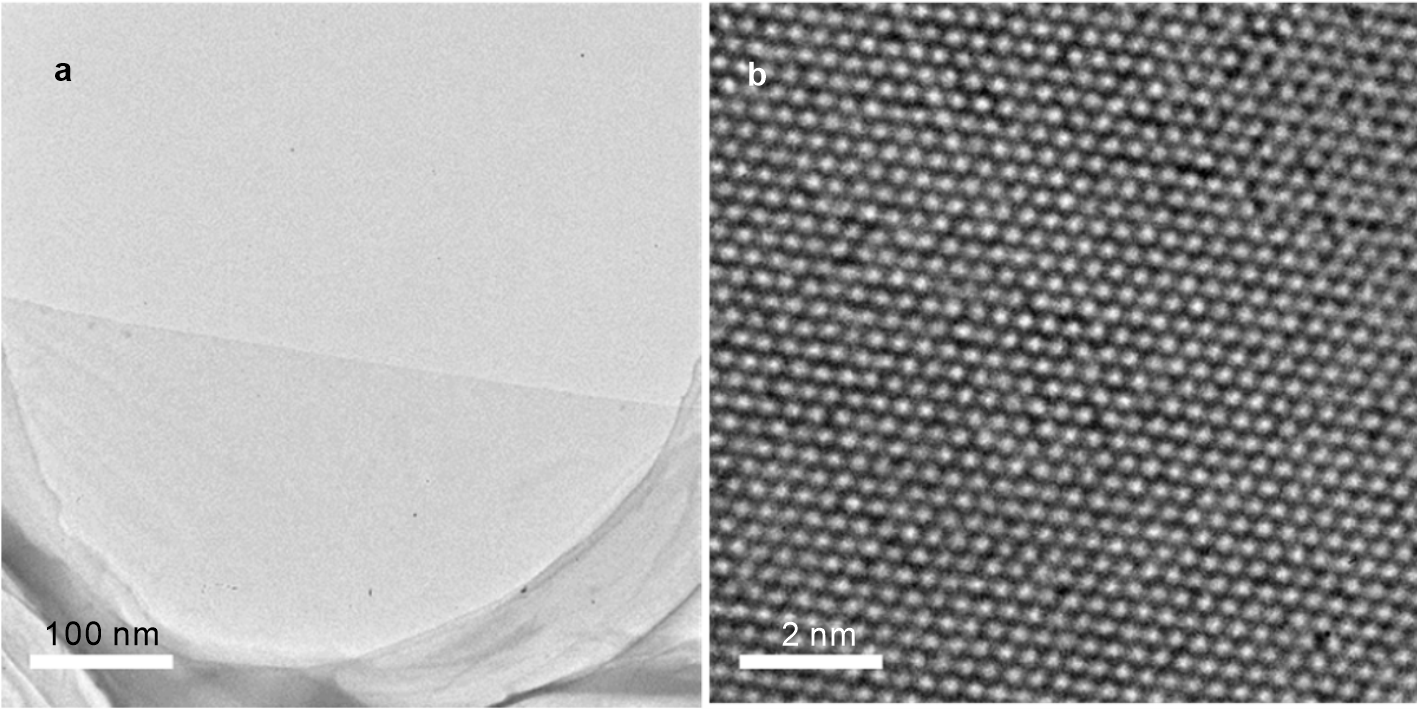


**Fig. S2**. Low-magnification image of a monolayer WSe_2_ at the flake edge. Scale bar in a: 100nm. (b) HRTEM image of a monolayer WSe_2_ monolayer. Scale bar: 2 nm.

High-resolution transmission electron microscopy (HRTEM) is also employed to investigate microstructure and crystallinity of the monolayer WSe_2_ crystals. The low-magnification and high-resolution TEM images of a monolayer WSe_2,_ are depicted in Fig. S2a and b, respectively. The low-magnification TEM image in Figs. S2a shows a corner of monolayer WSe_2_ crystal where the contrast is relatively uniform. Fig. S2b shows the HRTEM image, which clearly resolves the atomic lattice of monolayer WSe_2_.

# S2 Characterization of femtosecond laser written of WSe_2_ monolayer

The line width of each ring can be accurately controlled by the laser power. Due to the nonlinear multiphoton absorption, the data shows an exponential trend. We found the particle size shows random distribution and does not depend on the laser power.


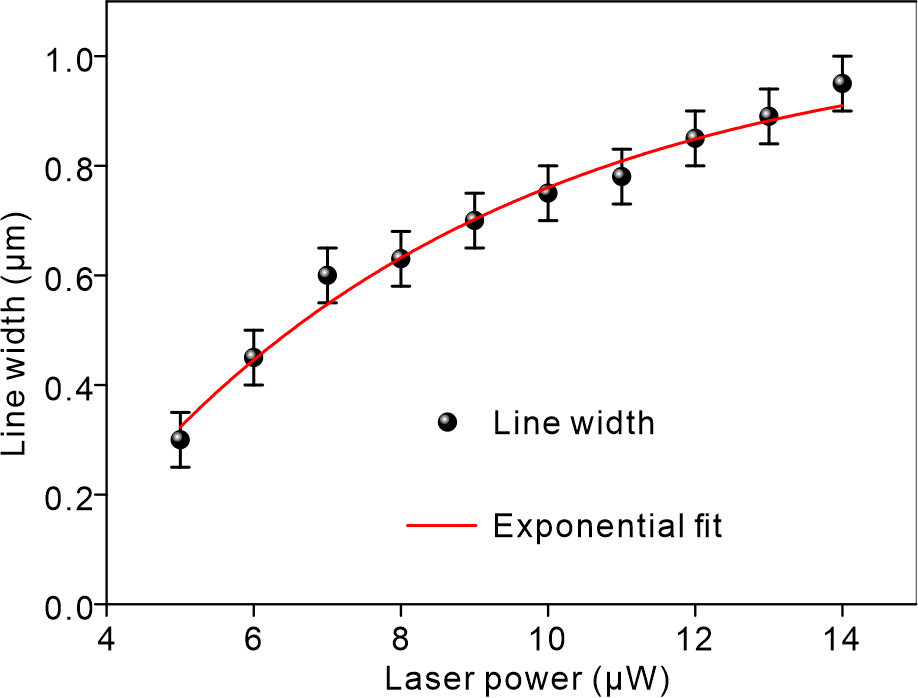


**Fig. S3** Linewidth versus laser power. An exponential function fits well with the linewidth confirming the multiphoton effect^8^.

# S3 Material characterization of femtosecond laser writing

## S3.1 Scanning micro-XPS characterization of femtosecond laser written area


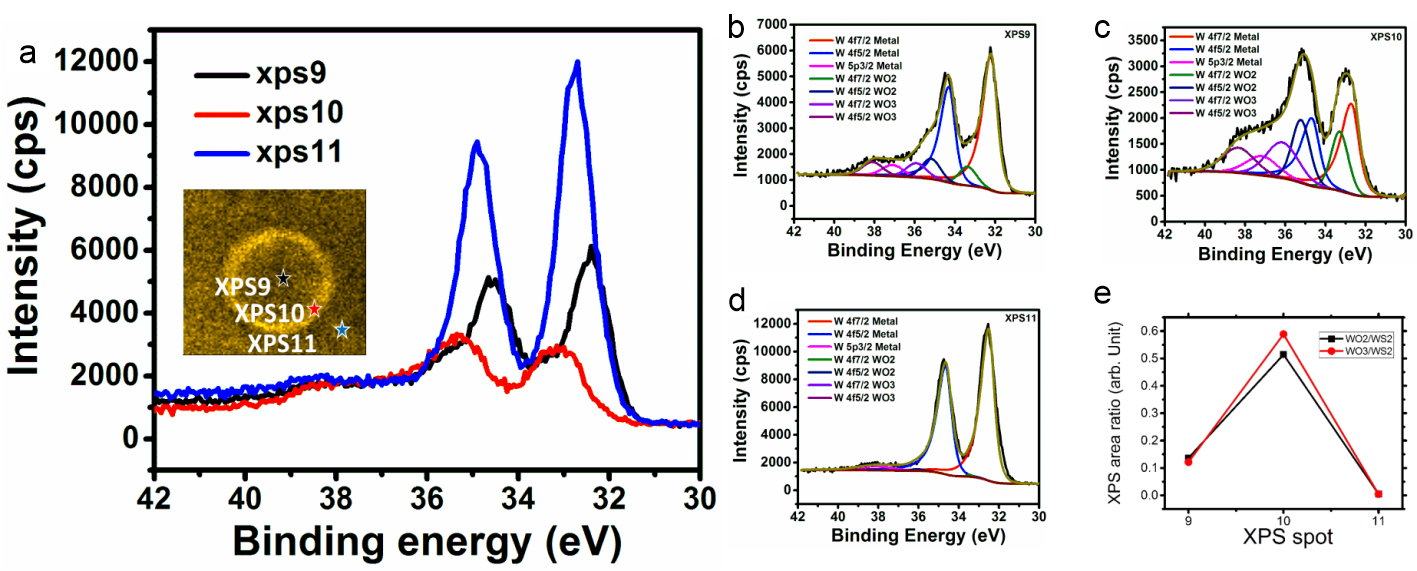


**Fig. S4** Spatial XPS characterization of the monolayer WS_2_ lens. A). Binding energy spectra of W from centre (xps 9), edge (xps 10) and the as-transferred WS_2_ (xps 11). Inset: Au 4f SPEM image generated by integrating the Au 4f photoelectron signal. The laser processed areas appeared brighter because the shielding effect of the Au 4f photoelectron resulted from WS_2_ film. (B-C) Experimental data and detailed fitting of each W binding energy spectra from xps 9, xps 10 and xps 11. (E) Summary of the WO_x_-to-WS_2_ intensity ratio from each spot.

Scanning XPS measurement was conducted at Synchrotron Radiation Research Center, Taiwan, China (SPEM end station of beamline 09A1). The soft X-ray beam (photon energy = 400 eV) was focused with Fresnel zone-plate optics to achieve a spatial resolution of 100 nm. Scanning photoelectron microscopy images were taken through scanning the sample holder on a piezo stage. The photon energy was routinely calibrated with the core-level line of Au at binding energy 84 eV. The overall energy resolution is better than 100 meV, and the experiments were conducted at room temperature.

To enhance the XPS signal, monolayer WS_2_ flakes were transferred onto a gold substrate before laser writing. Figure S4 shows the XPS spectra acquired from three different locations at the laser processed concentric ring on the WS_2_/Au film. The exact acquisition locations are marked in the Au 4f SPEM image (inset, image generated by integrating the intensity of Au 4f photoelectron), more specifically, xps 9 and xps10 stands for the positions of the centre and the edge of the concentric rings, xps 11 refers to the as-transferred WS_2_ monolayer. Detailed XPS spectra collected from these three spots were fitted and shown in Figs. S4 b-d. It is clear that in b and d, the predominant peaks are centred at ~33 and 35 eV, which mean most materials in these areas are still WS_2_, whereas in area marked xps 9, peaks corresponding to WO_2_ and WO_3_ are shown. Particularly, at position xps 10, the intensity of the W from the oxides almost equal to WS_2_. Figure S4d summarized the WO_2_/WS_2_ and WO_3_/WS_2_ ratios for each acquired location. It is clear that abundant WO_2_ and WO_3_ are generated after the laser processing. We anticipate that the formation of oxidized compounds is a general consequence after femtosecond laser treatment, which can also be applied on other van deer Waals dichalcogenides such as WSe_2_, MoS_2_, PtSe_2_ and PtS_2_.

## S3.2 AFM and Raman spectra of monolayer WSe_2_ material after milling.


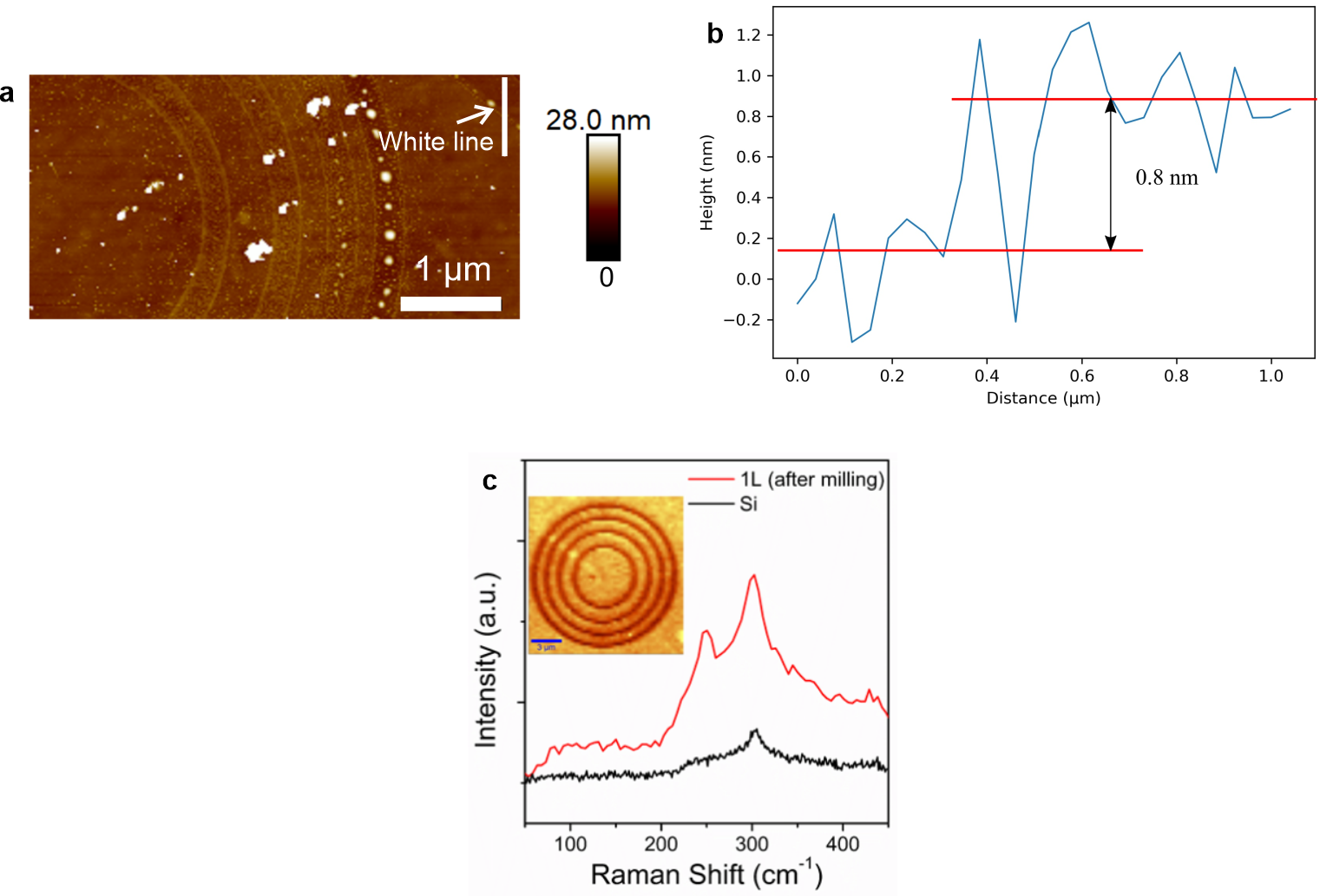


**Fig. S5** a) An AFM image of WSe_2_ lens. Scale bar: 1 μm. b) The cross-sectional profile along the white line in (a) at the edge of the single crystal WSe_2_. c) Raman spectra of monolayer WSe_2_ material after milling. Inset: 2D Raman image integrated with E^1^_2g_ band intensity.

In order to verify the monolayer nature of our sample, we extracted the WSe_2_/substrate step height from the AFM image shown in Fig. S5a. It can be seen that the thickness at the edge of single crystal WSe_2_ is ~0.8 nm (Fig. S5b), which is consistent with the thickness of TMD monolayer in the literature^1^.

The complete removal of the WSe_2_ material in the patterned area is further confirmed by Raman spectra (Fig. S5c) and E^1^_2g_ band intensity imaging (inset Fig. S5c). The shrink of the E^1^_2g_ at around 250 cm^-1^ and the rising peak at 308 cm^-1^ suggest that WSe_2_ is converted to WO_x_ after laser milling.

# S4 Dielectric constants of the WO_2_ and WSe_2_ materials

The complex refractive indices (complex permittivities) of WSe_2_ and WO_2_ materials are measured by using an spectral ellipsometer (M-2000 J.A. Woollam Co) and fitting by using the build-in software (Complete Ease) based on the Kramers-Kronig analysis^9^.

**Fig. S6** Real and imaginary part of the complex permittivity of WO_2_. At λ=633 nm, real permittivity of WO_2_ is 3.2, imaginary permittivity 8.73.


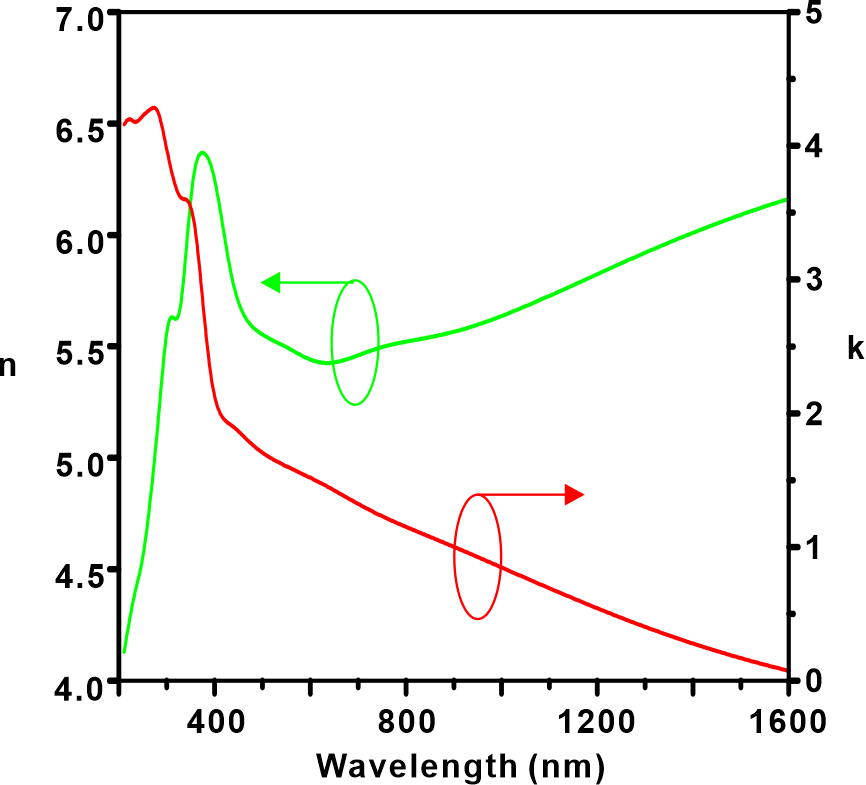


**Fig. S7** Refractive index and extinction coefficient of WSe_2_. At λ=633 nm, refractive index is 5.5, extinction coefficient is 1.5.

# S5 Numerical apertures of TMDC lenses versus different parameters

The plots of the numerical apertures (NAs) of TMDC lenses versus different parameters including the number of rings (*N*) and the radius of inner most ring (*a_1_*) are shown in Fig. S8, which is calculated by the following equation according to design.

$$NA=a_{N}/f$$

where the *a_N_* is the radius of the outmost ring and *f* is the focal length of the lens. For the number of rings, we fix the focal length at *f* = 9 μm. For the radius of inner most ring (*a_1_*), we fix the number of rings to be N=5,8,12, in this way, the focal length will vary according to the *a_1_*.


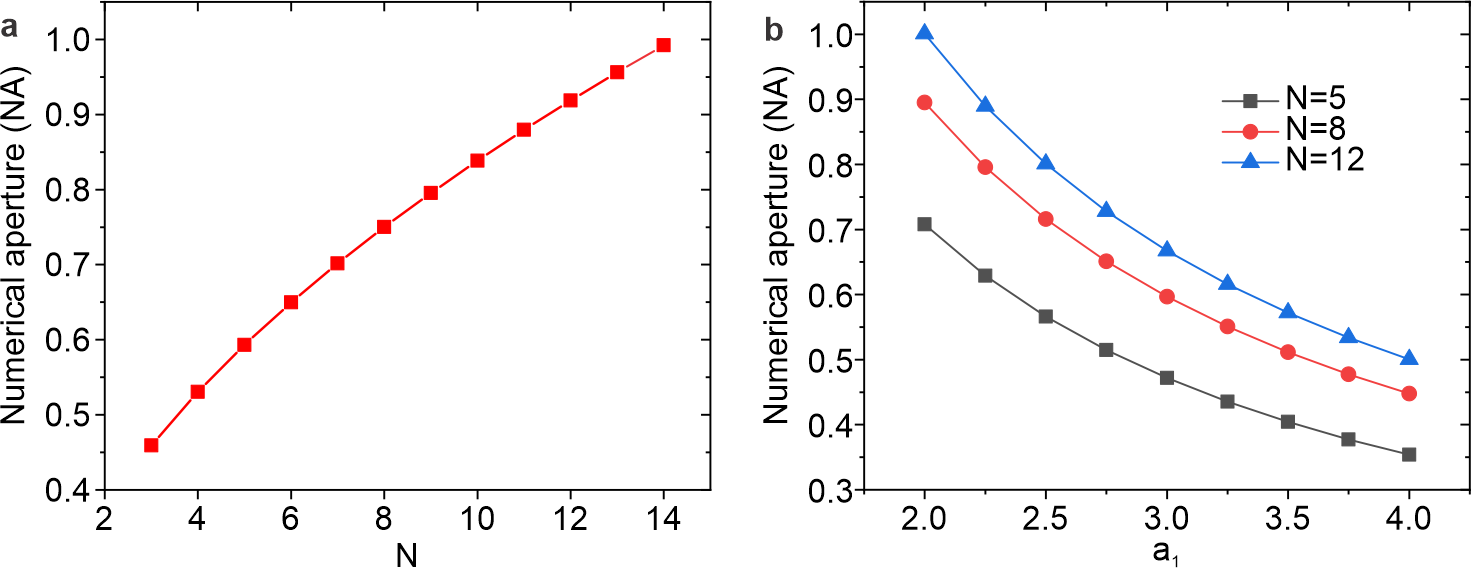


**Fig. S8** Numerical apertures (NAs) of TMDC lenses versus the number of rings (N) (a) and the radius of the inner most ring (*a_1_*) (b).

# S6 Characterization of the focusing properties of the monolayer WSe_2_ lens

A homemade imaging characterization system is built to study the performance of lenses, as schematically illustrated in Fig. S8. The cross-sectional distributions of the generated focal spots of the WSe_2_ lenses (which are attached to a SiO_2_ substrate) are captured using a CCD camera (Watec 902H3 SUPREME) equipped with a 100× objective (*NA* = 0.85) and a tube lens (f = 200 mm). The magnification rate of the 4f image system is 110. The lenses are illuminated by a collimated He-Ne laser at the wavelength of 633 nm. Therefore, each CCD pixel corresponds to a step size of 63 nm in the lateral direction. The 3D images of the focal spots are reconstructed by staking images captured at different len-to-objective distances, which are gradually adjusted using the 1D scanning stage with a step of ~10 nm. By normalizing the sensitivity and exposure time of the CCD camera, the lateral cross-sectional intensity distributions can be captured and the peak focusing intensities can be compared directly.


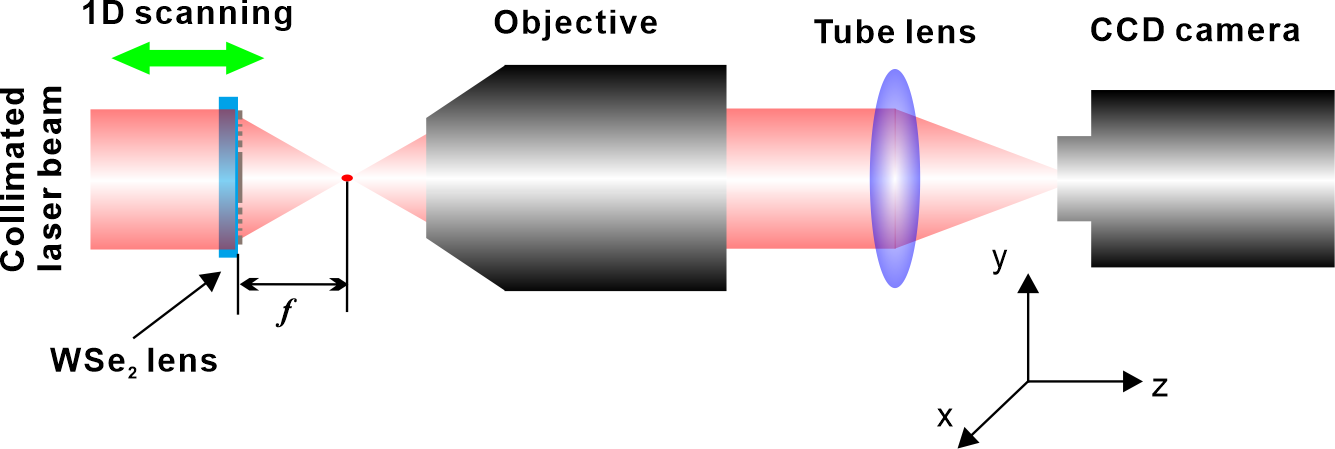


**Fig. S9** Optical setup for characterizing the focusing of monolayer WSe_2_ lens.

# S7 WSe_2_ lens fabricated by focused ion beam milling


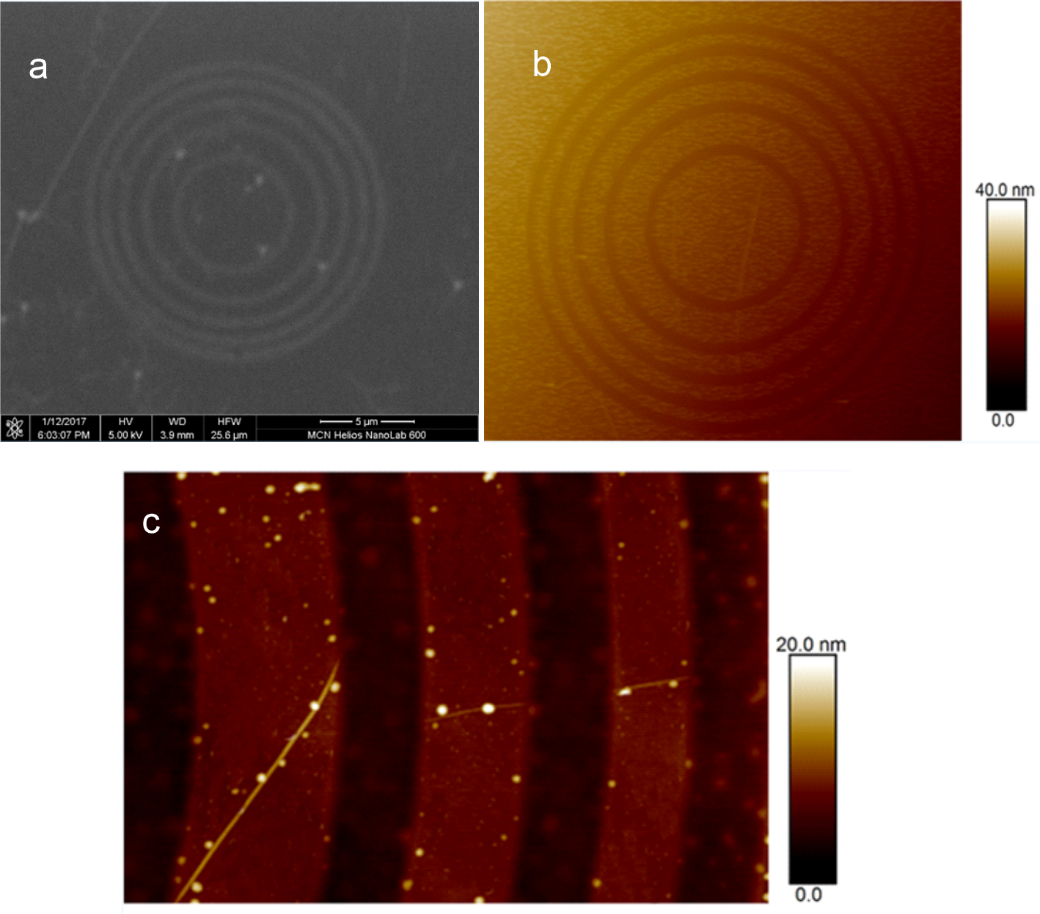


**Fig. S10** (a) SEM image of WSe_2_ lens fabricated by FIB, (b) AFM image of the WSe_2_ lens. (c) Zoom-in AFM image showing a limited number of nanoparticles. in the FIB-treated regions (with dark contrast).

# S8 WSe_2_ lens fabricated by femtosecond laser writing

Table S1 Summary of laser fabrication results.

| No. | Same laser condition | Thickness | Resolution in x-y plan (μm) | Z-resolution ((μm) | Focal length (μm) | Number of rings, *N* |
| --- | --- | --- | --- | --- | --- | --- |
| 1 | 14 µW | 7Å | 0.66 | 2.40 | 9.4 | 3 |
| 2 | 14 µW | 7Å | 0.48 | 2.10 | 9.9 | 4 |
| 3 | 14 µW | 7Å | 0.51 | 1.80 | 10.1 | 5 |
| 4 | 14 µW | 7Å | 0.50 | 1.70 | 9 | 6 |
| 5 | 14 µW | 7Å | 0.52 | 1.60 | 9.7 | 7 |
| 6 | 14 µW | 7Å | 0.49 | 1.50 | 9.3 | 8 |
| 7 | 14 µW | 7Å | 0.65 | 1.50 | 2.2 | 5 |
| 8 | 14 µW | 7Å | 0.58 | 1.70 | 4.1 | 5 |
| 9 | 14 µW | 7Å | 0.63 | 1.70 | 6 | 5 |
| 10 | 14 µW | 7Å | 0.50 | 1.80 | 7 | 5 |
| 11 | 14 µW | 7Å | 0.49 | 2.04 | 8.7 | 5 |
| 12 | 14 µW | 7Å | 0.47 | 1.60 | 10.7 | 5 |
| 13 | 14 µW | 7Å | 0.55 | 1.81 | 11.6 | 5 |
| 14 | 14 µW | 7Å | 0.52 | 1.78 | 12.2 | 5 |

# S9 Resolutions of lenses fabricated in different monolayer TMDC materials

The intensity distributions along the x-direction of lenses in different monolayer TMDC materials are shown in Fig. S10 and the full width at half maximum (FWHM) are plotted in Fig. S10 (e). As one can see in the figure, all the lenses are able to achieve subwavelength resolutions, suggesting the universality of the femtosecond laser fabrication method.


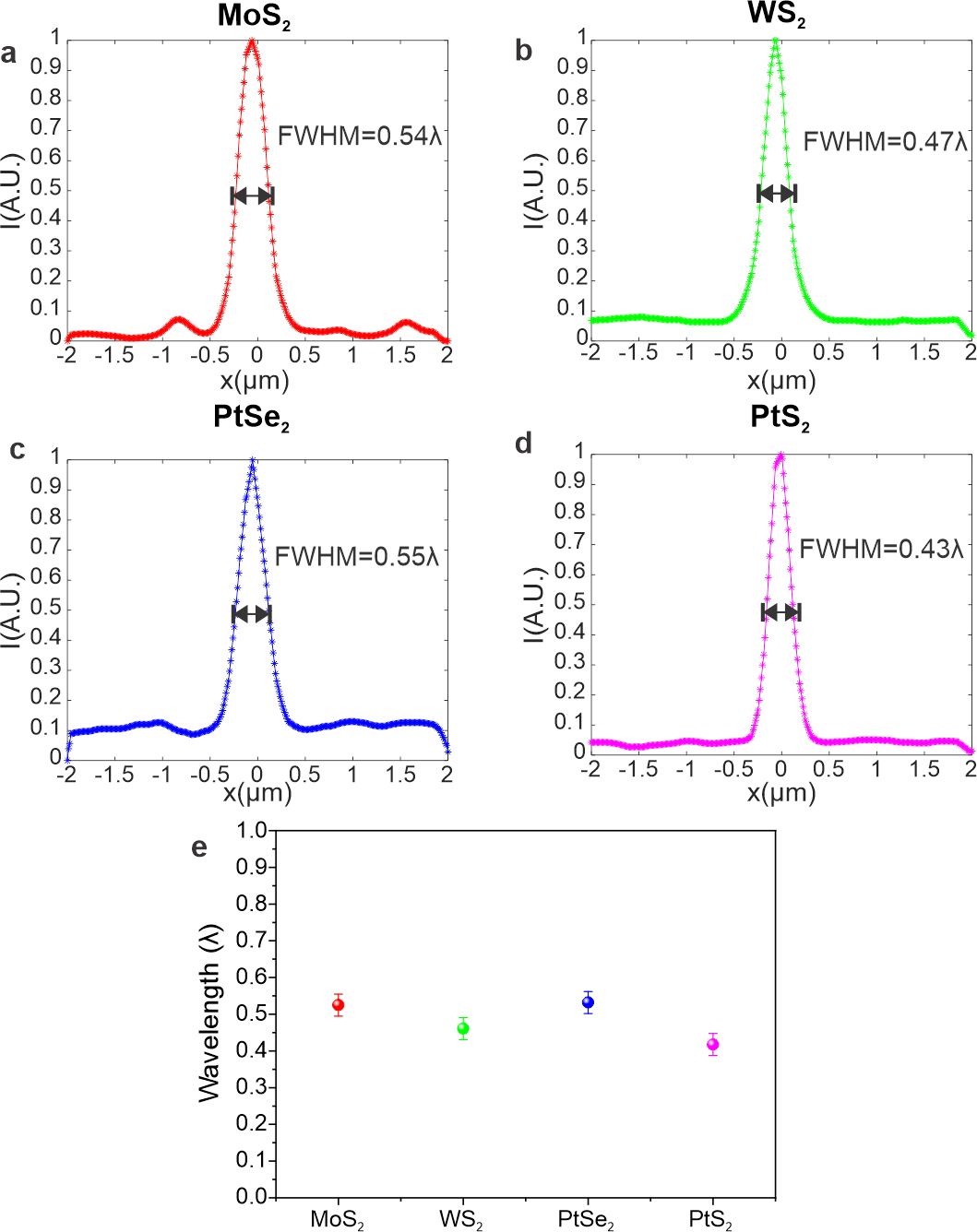


**Fig. S11** Resolutions of lenses fabricated in different TMDC materials. Intensity distributions along the x-axis direction of lenses made in MoS_2_ (a) WS_2_ (b) PtSe_2_ (c) and PtS_2_ (d). (e) Full width at half maximum of focal intensities of different monolayer TMDC materials.

# S10 Imaging using large-scale TMDC lens

In the design process, we consider only the first order diffraction as the main focal position, and *f_1_*=300 μm. According to our measurement, the *f_2_* is about 200 μm. The higher diffraction order results in a shorter focal length and image distance, because the diffraction angle of the higher order is larger than the lower order^10^ as shown in Fig. S11, thus resulting in a small magnification rate. In Fig. S11 one can see that the $f\propto cos\theta$.


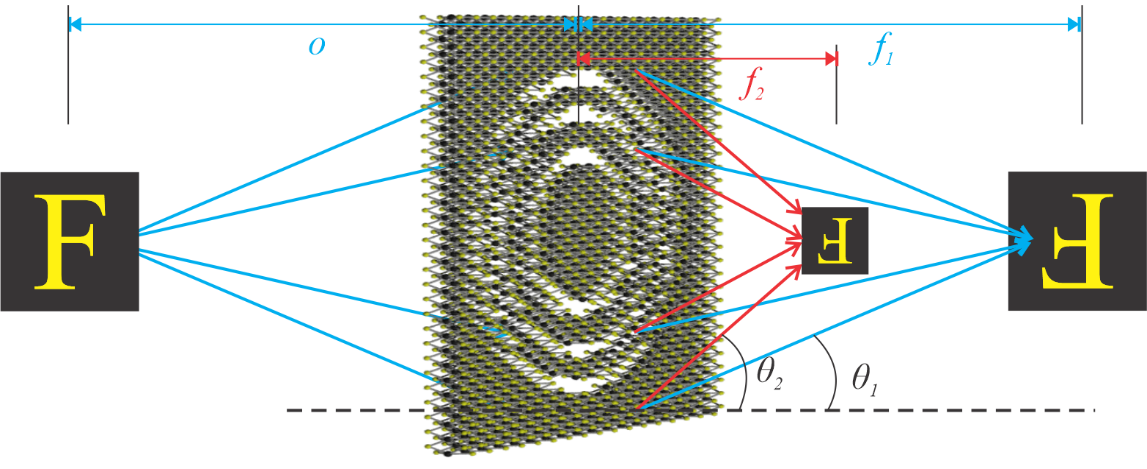


**Fig. S12** Schematic of the imaging scheme of the monolayer TMDC lens

The schematic of the imaging process is shown in Fig. S12, in which the images from the TMDC lens is further magnified by a 4f microscopic imaging system composing of an objective (NA=0.8, 100 ×) and a tube lens (*f*=200 mm) and collected using a CCD camera. The objective lens is scanned along the axial direction to obtain images at different positions. The object is illuminated by a white light source (Philips Essential 35W GU10 Dichroic Halogen Globe).


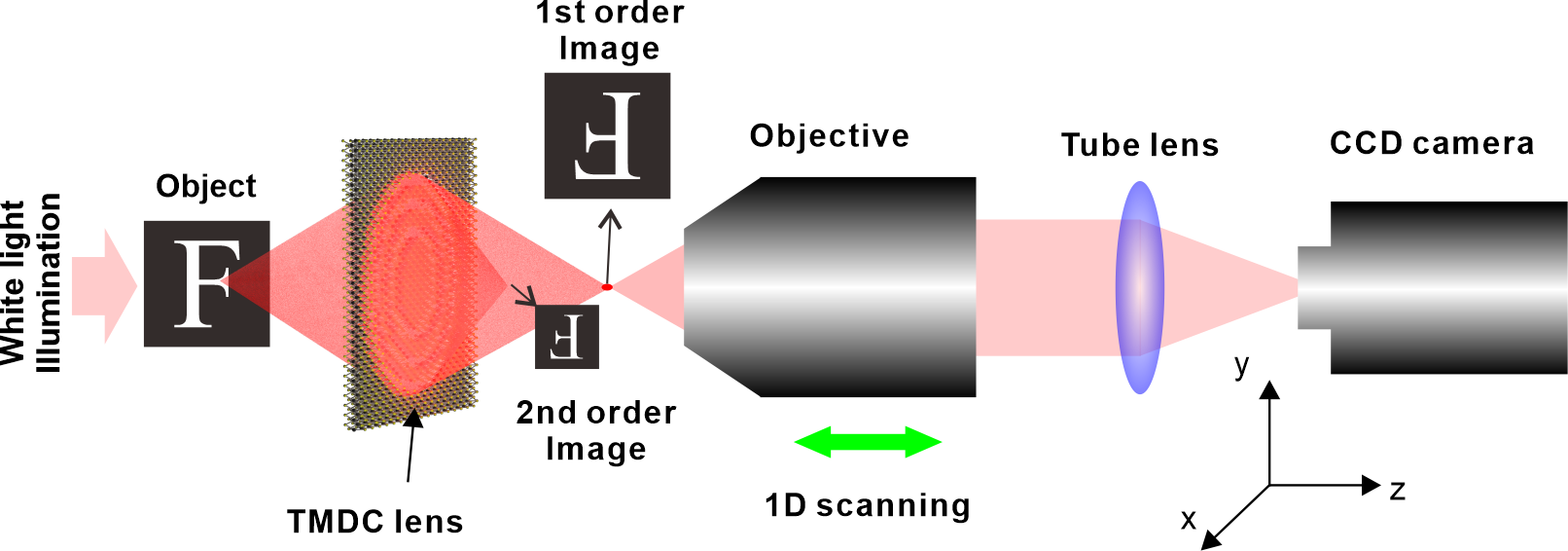


**Fig. S13** Schematic of the imaging experiment setup

Here we used two types of objects to demonstrate the imaging capability, namely the letter “F” and USAF standard target, which are used to test the imaging resolution of the lens. The objects are placed at the designed focal position of the lens, thus *o*=300 μm. Due to the high diffraction efficiency, both the first and the second order images can be clearly seen in the figure. From Eq. (3) in the manuscript, we can calculate the distance of the first order image is at 300 μm, which is the same as the object distance. The magnification rate is 1. In the meantime, the distance of the second order image is around 150 μm, and the magnification rate is 0.5. The minimal distance between bars in the USAF stand targe board in this experiment is 1.1 μm (Fig. 5f), which can be clearly identified in the first order image confirming the achievement of diffraction limited resolution. Furthermore, in the second order the distance between the bars is demagnified by 0.5 times, which gives a distance around 550 nm. Given the *NA* of the 4f imaging microscopic system is 0.8, the 550 nm feature cannot be clearly distinguished by the microscopic system. With a lens with multiple focal length, it is possible to achieve different zoom images with a single lens by putting the collecting images at different positions, which has not been demonstrated by other ultrathin flat lens according to our knowledge. Although currently the first order diffraction is stronger than the second order based on our design, it is possible to design a lens with arbitrary strength from different orders based on optimization method^11^ to meet application requirements.

# S11 Derivation of imaging rule of diffractive lenses based on geometric optics

Geometric optics can be considered as the limitation the Maxwell’s Theory of asymmetry that the wavelength is considered to be infinitely small^12^. The key of expressing the geometric optical phenomenon is to determine the optical path. A diffractive flat lens bends the ray trace based on diffraction. The ray-tracing schematic of the refractive and diffractive lenses are shown in Fig. S14.


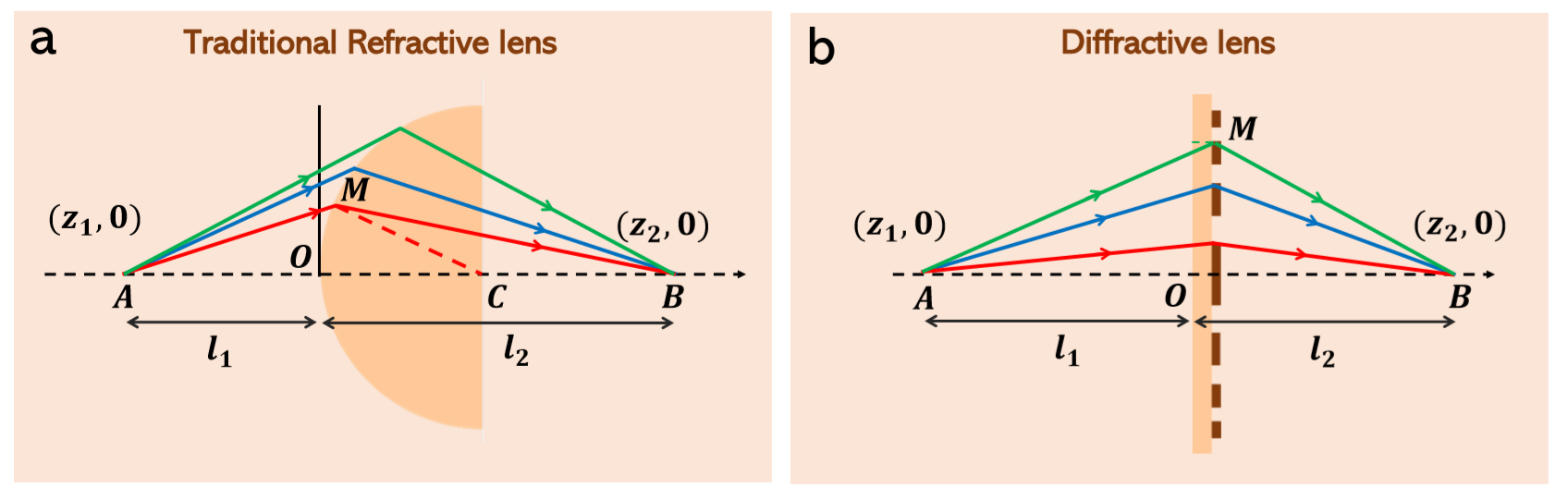


**Fig. S14** Comparison between (a) refractive lens and (b) diffractive lens.

In geometric optics, for paraxial region, the Eikonal equation can be regarded as:

$$\left( \nabla L \right)^{2}=n^{2} (2)$$

Where *L* is the optical path and *n* is the refractive index of the medium. The direction of $\nabla L$ is optical path direction. Take the square root of both side of the formula, we can get:

$$\nabla L=n\frac{dr}{ds} (3)$$

Where *r* is the position vector of any point on the light, $ds$ is the arc element of the extended light, the component equation of $x$ is:

$$\frac{\partial L}{\partial x}=n\frac{dx}{ds} (4)$$

As shown in Fig. S13,$l_{1}=\bar{AM}=\left[ x^{2}+\left( {z-z}_{1} \right)^{2} \right]^{1/2},l_{2}=\bar{MB}=\left[ x^{2}+\left( z_{2}-z \right)^{2} \right]^{1/2}$.

We can obtain the optical path L:

$$L=n_{1}\left[ x^{2}+\left( {z-z}_{1} \right)^{2} \right]^{1/2}+n_{2}\left[ x^{2}+\left( z_{2}-z \right)^{2} \right]^{1/2} (5)$$

In the case of a paraxial approximation, $z=x^{2}/2R$,$dx/ds=\left[ 1-\left( dz/ds \right)^{2} \right]^{1/2}\approx0$. The formula is taken into:

$$n_{1}\frac{x}{l_{1}}+n_{1}\frac{z}{l_{1}}\frac{x}{R}-n_{1}\frac{z_{1}}{l_{1}}\frac{x}{R}+n_{2}\frac{x}{l_{2}}+n_{2}\frac{z}{l_{2}}\frac{x}{R}-n_{2}\frac{z_{1}}{l_{2}}\frac{x}{R}=0 (6)$$

According to the paraxial approximation, $l_{1}={-z}_{1}，l_{2}=z_{2}$， and ignore the $x^{3}$，and finally obtain the equation:

$$\frac{n_{2}}{z_{2}}-\frac{n_{1}}{z_{1}}=\frac{n_{2}{-n}_{1}}{R} (7)$$

For the refractive lens, we can regard as: $f=2R, n_{2}=-n_{1}$, where $f$ is the focal length, and *R* is the radius of the refractive spherical lens. And then, we can obtain that:

$$\frac{1}{l_{1}}+\frac{1}{l_{2}}=\frac{1}{f} (8)$$

But in the diffraction theory model: $\left( R_{m}+\Delta\emptyset\right)-f=\frac{m\lambda}{2\pi}, n_{2}=-n_{1},\Delta\emptyset=x+z=\frac{m\lambda}{2\pi}$, where $\emptyset$ is the optical path from lens to focal plane.

The imaging rule of a diffractive lens can be defined as follows:

$$\frac{1}{l_{1}}+\frac{1}{l_{2}}=\frac{2}{f} (9)$$

# S12 Supplementary movies

Movie S1.

Femtosecond laser fabrication of lens structure in monolayer 2D material.

Movie S2.

Focal spot characterization of the fabricated lens.

Movie S3.

Imaging characterization using an object of letter “F”.

Movie S4.

Imaging characterization using an object of USAF standard target.

# Reference

1 Xu, Z. Q. *et al.* Synthesis and Transfer of Large-Area Monolayer WS2 Crystals: Moving Toward the Recyclable Use of Sapphire Substrates. *ACS nano* **9**, 6178-6187, doi:10.1021/acsnano.5b01480 (2015).

2 Peimyoo, N. *et al.* Nonblinking, intense two-dimensional light emitter: monolayer WS2 triangles. *ACS nano* **7**, 10985-10994, doi:10.1021/nn4046002 (2013).

3 Zhou, J. *et al.* A library of atomically thin metal chalcogenides. *Nature* **556**, 355-359 (2018).

4 Yim, C. *et al.* High-performance hybrid electronic devices from layered PtSe2 films grown at low temperature. *ACS nano* **10**, 9550-9558 (2016).

5 Huang, J.-K. & Li, L.-J. Large-Area Synthesis of Highly Crystalline WSe2 Monolayers and Device Applications. *ACS nano* **DOI: 10.1021/nn405719x**, doi:10.1021/nn405719x (2013).

6 Huang, J. *et al.* Large-area synthesis of monolayer WSe(2) on a SiO(2)/Si substrate and its device applications. *Nanoscale* **7**, 4193-4198, doi:10.1039/c4nr07045c (2015).

7 Zhou, H. *et al.* Large Area Growth and Electrical Properties of p-Type WSe2 Atomic Layers. *Nano letters* **15**, 709-713, doi:10.1021/nl504256y (2014).

8 Lin, H. *et al.* A 90-nm-thick graphene metamaterial for strong and extremely broadband absorption of unpolarized light. *Nature Photonics* **13**, 270-276, doi:10.1038/s41566-019-0389-3 (2019).

9 Morozov, Y. V. & Kuno, M. Optical constants and dynamic conductivities of single layer MoS2, MoSe2, and WSe2. *Applied Physics Letters* **107**, 083103, doi:10.1063/1.4929700 (2015).

10 Born, M. & Wolf, E. *Principles of optics: electromagnetic theory of propagation, interference and diffraction of light*. (CUP Archive, 2000).

11 Nie, Z.-Q. *et al.* Three-dimensional super-resolution longitudinal magnetization spot arrays. *Light: Science & Applications* **6**, e17032 (2017).

12 Gloge, D. & Marcuse, D. J. J. Formal quantum theory of light rays. **59**, 1629-1631 (1969).
